# Co-occurrence of Variants of *mcr-3* and *mcr*-*8* Genes in a *Klebsiella pneumoniae* Isolate From Laos

**DOI:** 10.3389/fmicb.2019.02720

**Published:** 2019-11-26

**Authors:** Linda Hadjadj, Sophie Alexandra Baron, Abiola Olumuyiwa Olaitan, Serge Morand, Jean-Marc Rolain

**Affiliations:** ^1^Aix Marseille Univ, IRD, MEPHI, Faculté de Médecine et de Pharmacie, Marseille, France; ^2^IHU Méditerranée Infection, Marseille, France; ^3^Assistance Publique des Hôpitaux de Marseille, Marseille, France; ^4^Institut des Sciences de l’Évolution, CNRS-IRD-UM2, CC065, Université Montpellier 2, Montpellier, France

**Keywords:** colistin, *mcr*, whole genome sequencing, resistance, epidemiology – descriptive

## Abstract

Colistin is considered as a last resort antibiotic. The re-use of this antibiotic highlighted the emergence of colistin resistance mediated by chromosomal and plasmidic resistance mechanisms. Five colistin-resistant *Klebsiella pneumoniae* strains from Laos and Thailand were analyzed by Next Generation Sequencing (NGS) approaches to determine their colistin resistance mechanisms. Antimicrobial susceptibility testing, conjugation and transformation were performed on these strains. Moreover, whole genome sequencing (WGS) combining Illumina (MiSeq) and Oxford Nanopore technologies (MinION) was realized to obtain closed genomes and plasmids. Resistome analyses as well as location of *mcr* genes and its genetic environments were done *in silico*. All five strains had colistin MIC of 32 mg/L and were positive for *mcr-3* variants including additionally positive for a *mcr-8* variant gene. The novel variants were named *mcr*-*3.21*, *mcr*-*3.26, mcr*-*3.28*, and *mcr-8.3* genes. The *mcr-3* variants genes were located on plasmids IncP1, IncFII, and IncI1 type, while *mcr-8.3* gene was found on an IncFII type plasmid. The genetic environment of *mcr*-*3.21* and *mcr-3.26* genes were composed of a composite transposon IS*Kpn40*- *mcr*-*3*-dgkA- IS*Kpn40.* Concerning *mcr-8.3* gene, a similar genetic environment of *mcr*-*8.1* gene surrounded by ISIX*2* and IS*903B* was observed. To the best of our knowledge, this is the first description of the novel variants *mcr*-*3.21*, *mcr*-*3.26, mcr-3.28* and *mcr*-*8.3* genes as well as the first study on co-occurrence of *mcr*-*3* and *mcr-8* genes. Spread and evolution of *mcr* genes should be monitored.

## Introduction

Antibiotic resistance is a major problem worldwide. This phenomenon has required adjustments in therapeutic approaches, including the use of powerful and broad-spectrum antibiotics, such as carbapenems against multi-drug resistant Gram-negative bacteria. The emergence of carbapenem resistant *Enterobacteriaceae* has led to the re-use of an old antibiotic: colistin (Biswas et al., 2012). This new therapeutic strategy promoted the apparition of colistin resistance mediated by chromosomal and plasmidic resistance mechanisms (Baron et al., 2016). In *Klebsiella pneumoniae*, *mgrB* inactivation appears to be the predominant mechanism of chromosomal resistance to colistin. Changes in amino acids in the two-component control systems PmrAB, PhoPQ, and CrrAB are also known to decrease colistin sensitivity by adding 4-amino-4-deoxy-l-arabinose and/or phosphoethanolamine to lipopolysaccharide (Baron et al., 2016). The *mcr-1* gene encoding a phosphoethanolamine transferase is the most widespread mechanism of colistin resistance mediated by a plasmid reported in the literature. To date, nine different plasmid-mediated colistin resistance genes have been described, noted from *mcr*-*1* to *mcr-9*, which are isolated from bacteria in human, animal and environmental samples (Liu et al., 2016; Yin et al., 2017; Wang et al., 2018; Carroll et al., 2019). Although *mcr-1* has been mainly reported in *Escherichia coli* from animal origins, *K. pneumoniae* can also harbor *mcr* genes (Yin et al., 2017). In a previous study, we isolated 32 colistin-resistant *K. pneumoniae* strains from human and animal samples collected in Laos, Thailand and France (Olaitan et al., 2014). 20 of these isolates had an inactivation of the *mgrB* gene but 12 strains had no colistin resistance mechanisms identified. In this work, we re-analyzed five of these strains by a Next-Generation Sequencing (NGS) approach to finally identify the mechanism of colistin resistance of these isolates.

## Materials and Methods

### Bacterial Strains and Antimicrobial Susceptibility Testing

Five colistin-resistant *K. pneumoniae* strains isolated in 2012 from stool samples of healthy humans from Laksip, Laos (LH102-A, LH94, LH375) and from Thailand (TH114, TH164) were reanalyzed by NGS approach to determine their colistin resistance mechanisms. Approval was given by the Ministry of Health Council of Medical Sciences, National Ethics Committee for Health Research (NHCHR), Lao PDR [no.51/NECHR] and by the Ethical Committee of Mahidol University, Bangkok, Thailand [no.0517.1116/661]. Each participant was informed and signed a consent form. Consents are kept at the Rodolphe Mérieux Laboratory in Laos, Vientiane, for Laos and at the Department of Helminthology, Faculty of Tropical Medicine, Mahidol University, Bangkok, for Thailand.

All strains had intact *mgr*B gene (Olaitan et al., 2014). Antimicrobial susceptibility testing (AST) was determined by minimum inhibitory concentrations (MICs) using microdilution with UMIC test (Biocentric, Bandol, France) for colistin and using the *E*-test method (bioMérieux, Marcy l’Etoile, France) for others antibiotics in accordance with EUCAST recommendations.

### Plasmid Transferability

To confirm *mcr* genes location biologically, conjugation was performed with an azide-resistant *E. coli* J53 strain. Selection of transconjuguants was done on MacConkey agar (Beckton Dickinson, Le Pont de Claix, France) supplemented with 120 mg/L sodium azide and 2 mg/L colistin. In case of unsuccessful conjugation, transformation of pure plasmid by electroporation method with Top10 electrocompetents *E. coli* (Thermo Fisher Scientific, Waltham, MA, United States) was performed and transformants were selected on LB agar (Beckton Dickinson, France) with 2 mg/L colistin. Plasmid curing was performed at high temperature on *K. pneumoniae* LH94 strain (Letchumanan et al., 2015). Then, the presence of *mcr* genes and plasmid types were controlled by standard PCR (Carattoli et al., 2005; García-Fernández et al., 2009; Rebelo et al., 2018; Wang et al., 2018).

### Genomic Analysis

All strains were sequenced using both MiSeq (Illumina Inc., San Diego, CA, United States) and MinION (Oxford Nanopore Technologies Inc., United Kingdom) technologies. Spades genome assembler was used to assemble long read sequencing data generated by Nanopore and short read data generated by Illumina (Bankevich et al., 2012). Genomes were annotated with RAST (Aziz et al., 2008), resistance gene using both ARG-ANNOT (Gupta et al., 2014) and blastN alignment, and the presence of plasmid was screened using PlasmidFinder software (Carattoli et al., 2014). oriTfinder was used to detect the presence of transfer-related modules of the sequenced plasmids: oriT region, relaxase, T4SS and T4CP (Li et al., 2018). The sequence type (ST) of *K. pneumoniae* strains was performed *in silico* by multilocus sequence typing (MLST) analysis using the MLST database (Larsen et al., 2012). The presence of known mutations conferring colistin resistance on the *mgrB, pmrA, pmrB*, *phoP, phoQ, crrA* and *crrB* genes has been also investigated *in silico* using both nucleic and amino acids alignments. Mutations of *mcr* genes have been verified by Sanger sequencing. The genetic environment of *mcr* genes has been reconstituted by comparing the sequence of genes surrounding the *mcr*-like genes against the NCBI database, using blastX parameter. These environments were also compared to those of *mcr*-*3.1* (KY924928), *mcr*-*3.11* (MG552133) and *mcr*-*8.1* (MG736312) genes. The plasmid pKP91 (MG736312) carrying the first *mcr-8.1* gene described in the literature was compared with the plasmid pLH94-8 (CP035204) of the strain LH94 that harbor a *mcr*-*8.3* gene using a pairwise comparison of their Average Nucleotide Identity based on Blast (ANIb) on Jspecies software (Richter et al., 2016). CGViewServer was used to represent the complete sequences of plasmids carrying *mcr* genes (Stothard et al., 2017).

**FIGURE 1:**
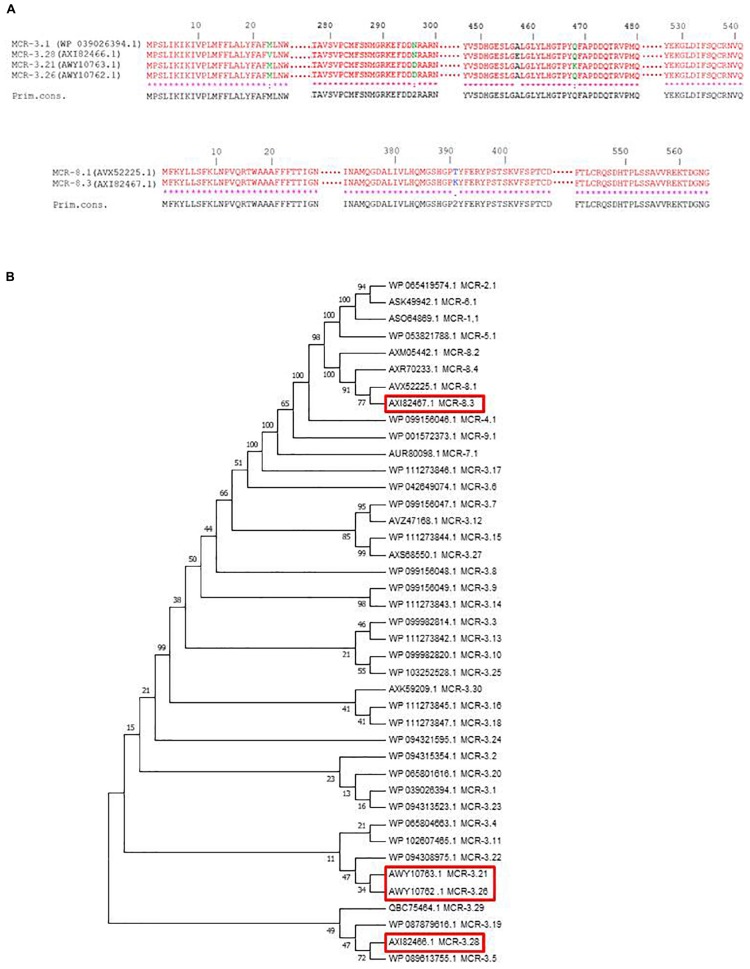
**(A)** Amino acid sequence alignments of the new MCR-3 and MCR-8 variants using Clustal W on NPS@ website. **(B)** Phylogenetic tree of MCR variants, including MCR-3 and MCR-8 variants. Sequences were aligned using MUSCLE and phylogenetic interferences were obtained using the neighbor-joining method within the MEGA 7 software. Numbers at the nodes are percentages of bootstrap values obtained by repeating the analysis 500 times to generate a majority consensus tree. Evolutionary distances were computed using the Poisson correction method and are expressed in units of the number of amino acid substitutions per site.

Genomes of LH102-A, LH94, LH375, TH114, and TH164 strains have been submitted to GenBank under accession numbers CP035194-CP035195, CP035202-CP035206, CP035196-CP035201, CP035207-CP035209, and CP035210-CP035213, respectively (Table 1). Novel variants of *mcr*-*3* and *mcr*-*8* genes were detected and assigned by NCBI (Partridge et al., 2018) as *mcr*-*3.21* (AWY10763), *mcr*-*3.26* (AWY10762), *mcr*-*3.28* (AXI82466) and *mcr*-*8.3* (AXI82467) (Figures 1A,B).

**TABLE 1:** Characteristics of *Klebsiella* spp. strains analyzed in this study.

**Strains**	**Genome size (bp)**	**GC%**	**ST**	**Sensitive to (MIC mg/L)**	**Resistant to (MIC mg/L)**	**ARGs**	**Plasmid type**	**Conjugal modules predicted**	**Accession number**
*K. pneumoniae* LH102-A	5,213,480	57.0	87	CRO (0.023), ERT (0.006), IMP (0.25), FF (32), AK (2), GN (0.38), SXT (0.064), CIP (0.047)	AMX (>256), AMC (4), CT (32)	*bla*_SHV–__27_	/	/	CP035194
						*bla*_TEM–__1__B_, ***mcr-3.28***	IncP1 (pLH102-A)	oriT, Relaxase, T4SS, T4CP	CP035195
*K. pneumoniae* LH94	5,709,714	57.0	39	CRO (0.032), ERT (0.006), IMP (0.19), FF (32), AK (2), GN (0.38)	AMX (>256), AMC (4), SXT (>32), CIP (1), CT (32)	*bla*_SHV–__11_	/	/	CP035202
						*bla*_TEM–__1__*B*_, *qnrS1, aadA1, aadA2, floR, cmlA1, sul3, sul2, dfrA12*, ***mcr-8.3***	IncFII (pLH94-8)	oriT, Relaxase, T4SS^∗^, T4CP	CP035204
						***mcr*-*3.21***	IncI1 (pLH94-3)	oriT, Relaxase, T4SS, T4CP	CP035205
*K. pneumoniae* LH375	5,372,573	57.3	1315	CRO (0.032), ERT (0.008), IMP (0.25), FF (24), AK (2), GN (0.38), SXT (0.064)	AMX (>256), AMC (4), CIP (1), CT (32)	*bla*_SHV–__1_	/	/	CP035196
						***mcr*-*3.26***	IncFII (pLH375-3)	oriT, T4SS	CP035200
						*bla*_TEM–__1__*C*_, *qnrS1, aadA2, Inu(F), floR, tetA*	IncR (pLH375-2)	/	CP035198
*K. quasipneumoniae* TH114	5,242,749	57.7	1321	CRO (0.032), ERT (0.008), IMP (0.25), FF (32), AK (2), GN (0.38), CIP (0.032)	AMX (>256), AMC (4), SXT (>32), CT (32)	*bla*_OKP–B–__7_	/	/	CP035207
						***mcr*-*3.21***	IncI1 (pTH114-3)	oriT, Relaxase, T4SS, T4CP	CP035208
						*bla*_TEM–__1__B_, *aadA2, floR, sul2, tetA, dfrA12*	IncR (pTH114-1)	oriT	CP035209
*K. pneumoniae* TH164	5,656,506	57.3	873	CRO (0.023), ERT (0.006), IMP (0.25), FF (16), AK (2)	AMX (>256), AMC (4), SXT (>32), CIP (1), GN (48), CT (32)	*bla*_SHV–__27_	/	/	CP035210
						***mcr*-*3.21***	IncI1 (pTH164-3)	oriT, Relaxase, T4SS, T4CP	CP035213
						*bla*_TEM–__1__B_, *qnrS1, aadA1, aadA2, aac(3)-IId, floR, cmlA1, sul3, sul2, tetA, dfrA12*	IncR (pTH164-1)	oriT	CP035212

*MIC, minimum inhibitory concentration; ARGs, antibiotic resistance genes; ST, sequence type; AMX, amoxicillin; AMC, amoxicillin-clavulanic; CT, colistin; CRO, ceftriaxone; ERT, ertapenem; IMP, imipenem; FF, fosfomycin; AK, amikacin; GN, gentamicin; SXT, trimethoprim-sulfamethoxazole; CIP, Ciprofloxacin; ^∗^, incomplete.*

## Results

All *K. pneumoniae* strains had a colistin MIC at 32 mg/L (Table 1). They remain susceptible to ceftriaxone, ertapenem, imipenem, fosfomycin and amikacin but were resistant to amoxicillin and amoxicillin-clavulanic acid. Resistance to trimethoprim-sulfamethoxazole, ciprofloxacin and gentamicin was inconstantly observed (Table 1). The resistome analysis of these strains indicated the presence of genes encoding resistance for beta-lactams (*bla*_SHV,_ *bla*_OKP–B,_ *bla*_TEM_), aminoglycosides (*aadA)* sulphonamides (*sul)*, fluoroquinolones (*qnrS1)*, fosfomycin, phenicol (*floR)*, tetracycline (*tetA)*, trimethoprim (*dfrA)*, and colistin *(mcr)* (Table 1).

Genome size of the five *Klebsiella* spp. strains ranged from 5,213,480 to 5,709,714 bp with chromosomes and plasmids included for each strain. Blast analysis of RpoB protein and the specific presence of the *bla*_OKP–B–__7_ gene instead of *bla*_SHV_ gene allow to reclassify TH114 isolate as a *Klebsiella quasipneumoniae* strain (Table 1). According to the MLST analysis, each strain belonged to different sequence types (ST39, ST97, ST873, ST1315, and ST1321).

We observed no disruption or known mutations in the *mgrB* gene or in the different two-component systems *pmrAB*, *phoPQ*, and *crrAB* of these five genomes. However, *K. pneumoniae* LH102-A and LH375 carried a *mcr-3.28* and a *mcr*-*3.26* gene, respectively. The strains LH94, TH164 and TH114 harbored a *mcr*-*3.21* gene, but LH94 isolate also carried *mcr*-*8.3* gene (Table 1). Compared to *mcr-3.1* gene, *mcr-3.21* displayed two amino acids substitution (N296D and Q468K), while *mcr-3.26* had only one amino acid change (N296D). For the *mcr-3.28* gene, two mutations (M23V and A457E) were identified. The *mcr-8.3* gene is distinguished from *mcr-8.1* by an amino acid substitution T391K (Figures 1A,B). In each strain, the *mcr*-*3* gene was present on one plasmid but lastly only conjugate in LH102-A, LH375 and TH114 strains. The transconjuguants and transformants isolates had a colistin MIC of 2 mg/L and were susceptible to antibiotics previously tested except for amoxicillin in a transconjugant LH102-A strain which was consistent with the presence of a *bla*_TEM–__1__*B*_ one the plasmid harboring the *mcr* gene (Table 1). The *mcr-8* and *mcr-3* genes of the LH94 strain were not found in the original strain after curing plasmid. In addition, the *mcr-8* gene was not detected in transformants of the LH94 strain carrying the *mcr-3* gene, which confirms its presence on another plasmid. The presence of potential transfer-related modules, including the origin of transfer (*oriT*) region, the relaxase gene, T4CP gene and the type IV secretion system gene cluster (T4SS) were detected in some strains (Table 1).

**FIGURE 2:**
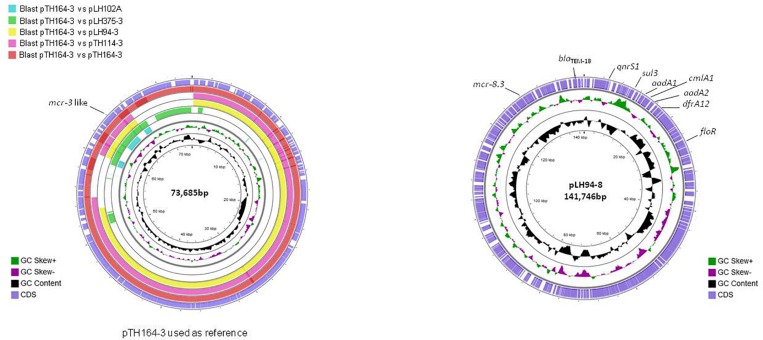
Circular representation of the five complete *mcr-3* plasmids **(left)** and the *mcr-8* plasmid **(right)**. Figure constructed using CGView software.

**FIGURE 3:**
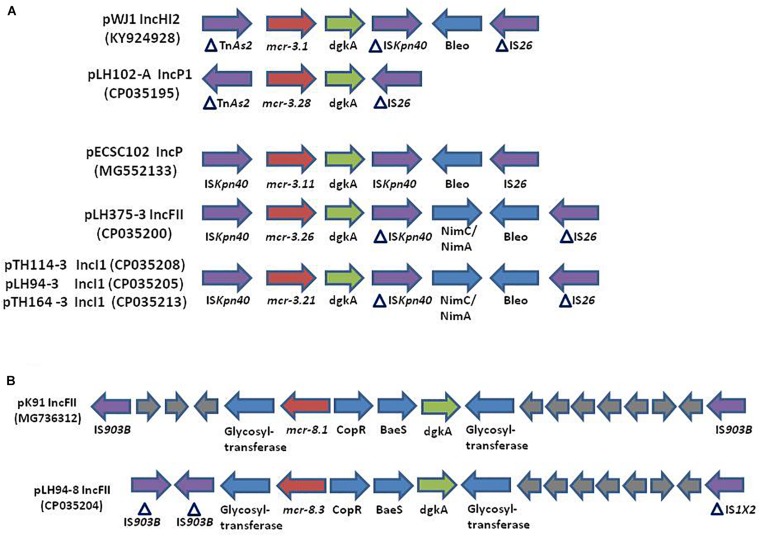
(Not to scale) **(A)** Comparison of the genetic environment of the *mcr-3* gene in plasmids: pWJ1 (KY924928), pLH102-A (CP035195), pECSC102 (MG552133), pLH375-3 (CP035200), pTH114-3 (CP035208), pLH94-3 (CP035205), and pTH164-3 (CP035213). **(B)** Comparison of the genetic environment of the *mcr-8* gene in plasmid pK91 (MG736312) and in the plasmid of strain LH94 carrying *mcr*-*8.3* gene: pLH94-8 (CP035204). HP, hypothetical protein, IS, insertion sequence, Tn, transposon.

The reconstitution of plasmids was achieved using reads from both Miseq and Minion sequencer. The three *mcr-3.21* genes were carried by an IncI1 plasmid type (LH94, TH114 and TH164) whereas the *mcr-3.28* gene was harbored by an IncP1 (LH102-A) and the *mcr3.26* by an IncFII (LH375). The *mcr-8.3* gene was also found on an IncFII plasmid type (LH94) (Table 1). Plasmid structure of the five plasmids carrying *mcr-3* genes and the plasmid containing the *mcr-8* gene was represented in Figure 2. Plasmids LH94, TH114, and TH164 belonging to IncI1 plasmid type were very similar, except for TH164 that carried more transposases genes around *mcr* gene. Although the close genetic environment of *mcr* genes was very similar between the five plasmids, the LH375 and LH102A ones belong to other plasmid type and so were different from the IncI1 plasmids. The genetic environments of *mcr*-*3.21* and *mcr*-*3.26* genes in strains TH114, LH94, TH164, and LH375 were similar and composed of a composite transposon IS*Kpn40*- *mcr*-*3.21*-*dgkA*- IS*Kpn40* such as *mcr*-*3.11* gene (Figure 3A; Xiang et al., 2018). Concerning the *mcr*-*3.28* gene, it was framed by two mobile elements as follow: Tn*As2*- *mcr*-*3.28*- dgkA-IS*26.* For *mcr*-*8.3* gene, a similar genetic environment of *mcr*-*8.1* gene was observed (Figure 3B). It was interesting to note the presence of two genes annotated as a response regulator transcription factor CopR for the first one (EQH49_28300) and as a HAMP domain-containing histidine kinase BaeS for the second one (EQH49_28305). The combination of these two protein families generally constitutes a two-component system. These systems are often involved in colistin resistance in *Enterobacteriaceae* (*pmrAB*, *crrAB* or *phoPQ*). The presence of a two-component system on a plasmid carrying the *mcr-8* gene requires further analysis to understand its role in colistin resistance. The plasmids pLH94-8 (CP035204) and pKP91 (MG736312) harboring *mcr-8* gene shared 95.42% of identity.

## Discussion

Following plasmid-mediated colistin resistance gene *mcr-1*, the *mcr-3* gene is the most widely described variants found in the literature, while *mc*r-*2*, and *mcr*-*4* to *mcr*-*8* remain uncommon. The MCR-3 protein shares only 32.5% amino acid with MCR-1 which makes impossible its detection with previously described *mcr-1* primers (Yin et al., 2017). Since its first description in 2017, thirty variants of *mcr*-*3* gene have been deposit in NCBI and this gene has been observed in different countries and in different strains such as *E. coli*, *Aeromonas* spp., and *K. pneumoniae* (Figure 1B; Yin et al., 2017; Fukuda et al., 2018; Shen et al., 2018).

The presence of *K. pneumoniae* strains carrying the *mcr*-*3* gene in samples from humans in Thailand has already been described (Yin et al., 2017) but never in Laos. Similarly to the transposon Tn*6330* (*I*S*Apl1*-*mcr*-*1*-ORF-IS*Apl1*), a composite transposon IS*Kpn40*- *mcr-3*-*dgkA*- IS*Kpn40* has been reported in several type of plasmids (Xiang et al., 2018). It is known the integration of this type of transposon into a variable region is mediated by insertion sequences (IS), that are then lost to stabilize the integration of the transposon (Snesrud et al., 2018). The genetic environment of our *mcr*-*8.3* gene was similar to that of the *mcr-8.1* gene in the reference plasmid pK91 which was isolated from a swine fecal sample in China (Wang et al., 2018). However, unlike plasmid pK91, the pLH94-8 plasmid also harbors other antibiotic resistance genes. The co-occurrence of different antibiotic resistance genes could explain the presence of two *mcr* genes in a same isolate (Bi et al., 2015). Indeed, this *mcr-8* gene could have been selected because it was harbored by a plasmid that also conferred resistance to beta-lactam, aminoglycoside, sulphonamide, fluoroquinolone, phenicol and trimethoprim.

The first description of the *mcr*-*8* gene was done in *K. pneumoniae* strains isolated from animals and human in 2016 in China. This gene was found on a conjugative IncFII type plasmid (Wang et al., 2018). To date, the *mcr-8* gene and its variants have only been described in China in *Stenotrophomonas* spp., *Raoultella ornithinolytica* and *K. quasipneumoniae* from animal and environmental origins (Li et al., 2019; Wang et al., 2019; Yang et al., 2019). Since MCR-8 had only 30.26 to 39.96% identity with other MCR, it is possible that the prevalence of *mcr-8* gene is underestimated. Reanalysis of previous works will probably respond to this question. In our study, the detection of a *mcr*-*8.3* gene in a *K. pneumoniae* strain isolated from a 2012 sample from Laos raises the question of the spread of this gene. Moreover, the emergence of novel variants for *mcr*-*3* and *mcr*-*8* gene indicates that this gene has evolved over time. Furthermore, co-location in the same strain on different plasmids encoding different *mcr* genes and others antibiotic resistance genes increases the risk of spread of colistin resistance (Long et al., 2019). Antibiotic resistance genes and their transferability are a major risk for human health. The “One health” approach promoting systemic coordination of three sectors: environment, animals and humans could be beneficial for the effective control of global antibiotic resistance problems (Ryu et al., 2017).

To the best of our knowledge, this is the first study describing the novels variants *mcr*-*3.21*, *mcr-3.26, mcr-3.28, mcr-8.3* genes and demonstrating the co-existence of MCR-3 and MCR-8 in a *K. pneumoniae* isolate from Laos.

## Data Availability Statement

The datasets analyzed in this manuscript are publicly available on GenBank. Requests to access the datasets should be directed to linda.hadjadj@uni-amu.fr.

## Author Contributions

LH wrote the manuscript, performed the experiments, and analyzed the data. SB helped for bioinformatics analysis and to draft the manuscript. AO isolated the strains and helped to draft the manuscript. SM was responsible for the collection of samples and helped to draft the manuscript. J-MR conceived the study, participated in designing and coordination, and helped to draft the manuscript. All authors read and approved the final draft of the manuscript.

## Conflict of Interest

The authors declare that the research was conducted in the absence of any commercial or financial relationships that could be construed as a potential conflict of interest.
